# N-Heterocyclic carbene-based gold etchants

**DOI:** 10.3762/bjnano.14.71

**Published:** 2023-08-21

**Authors:** Robert B Chevalier, Justin Pantano, Matthew K Kiesewetter, Jason R Dwyer

**Affiliations:** 1 Department of Chemistry, University of Rhode Island, 140 Flagg Road, Kingston, RI, 02881, USA

**Keywords:** gold etchant, microfabrication, N-heterocyclic carbenes, self-assembled monolayer (SAM), thin films

## Abstract

N-Heterocyclic carbenes (NHCs) are an emerging alternative to thiols for the formation of stable self-assembled monolayers (SAMs) on gold. We examined several different species that have been used to produce NHC-based monolayers on gold, namely 1,3-diisopropyl-5-nitrobenzimidazolium iodide, 1,3-diisopropyl-5-nitrobenzimidazolium hydrogen carbonate, bis(1,3-diisopropyl-5-nitrobenzimidazolium)gold(I) iodide, and 1,3-diisopropyl-5-nitrobenzimidazole-2-ylidene. Contrary to expectation, solutions containing the first two species in tetrahydrofuran and dichloromethane caused visible loss of gold from thin-film-coated glass slides. The use of toluene solutions of all species resulted in no apparent dissolution of gold. We present scanning electron micrographs and elemental imaging analyses by energy dispersive X-ray spectroscopy to examine the effect of solutions of each species on the gold film. This work highlights the risk of unwanted etching during some routes to NHC-based surface functionalization but also the potential for deliberate etching, with the outcome determined by choice of chemically synthesized organic species and solvent.

## Introduction

Self-assembled monolayers (SAMs) are a fixture of materials science. Thiols continue to dominate as the most prevalent choice of SAM constituents on gold [1–11]. They benefit from a long history of widespread use and general ease of adoption. Unfortunately, the gold–sulfur bond can limit the range of conditions that the thiol-linked monolayer can be exposed to, prompting the development of alternative surface linking chemistry [12]. N-Heterocyclic carbene (NHC)-based monolayers have received increasing attention for their reported stability under a variety of harsh conditions [11,13–16]. Indeed, an NHC-based monolayer has even been shown to protect against thiol displacement on gold surfaces [13]. Thus, in spite of the challenges that can be presented by NHC synthesis [17], they offer appeal even in light of the more general commercial availability of a wide range of thiols. The covalent attachment of NHCs to gold and the properties of the corresponding monolayers have been studied using conventional surface science techniques under ultrahigh-vacuum conditions [13–14]. NHC monolayers have also been used in applications such as surface-enhanced Raman spectroscopy and surface plasmon resonance in solution-phase samples [15,18–22]. In these works, the NHC monolayer films were formed using several different approaches and preparations [16]. Indeed, a recent feature article highlights four methods to prepare NHC films [16]. For example, Crudden and co-workers have presented two different solution-based surface functionalization routes. One involved the deprotonation of a benzimidazolium precursor in toluene under air-free conditions. The other was in methanol and took advantage of the equilibrium between a benzimidazolium hydrogen carbonate salt and free carbene with water and carbon dioxide so that there was no need for a strong base for deprotonation or for air-free conditions [13–14]. Camden and co-workers have used CO_2_ adducts of benzimidazolium to produce NHC films by melting the solid CO_2_ adduct directly onto a gold surface under vacuum. They have also reported the use of a benzimidazolium gold complex in dichloromethane (DCM) that readily functionalizes gold nanoparticles (AuNPs) in aqueous solution [15,19].

**Figure 1:**
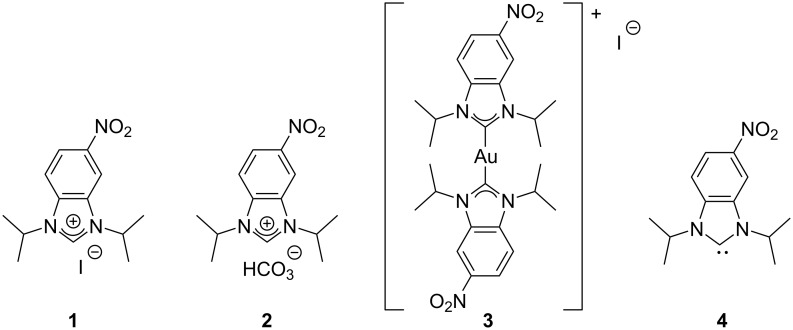
Four species were examined, namely 1,3-diisopropyl-5-nitrobenzimidazolium iodide (**1**), 1,3-diisopropyl-5-nitrobenzimidazolium hydrogen carbonate (**2**), bis(1,3-diisopropyl-5-nitrobenzimidazolium)gold(I) iodide (**3**), and 1,3-diisopropyl-5-nitrobenzimidazole-2-ylidene (**4**).

In our work using solution-based approaches to form NHC monolayers on gold thin films, we observed a loss of gold from our substrates. It is clearly undesirable that an effort to form a stable monolayer could result in removal of underlying material, but similarly undesirable effects have been reported in the gold–thiol SAM literature. For example, gold–sulfur interactions can lead to a weaker bonding of gold surface atoms to the bulk. This, in turn, can promote surface mobility of gold across a sample, allowing for the formation of herringbone structures, pits, and step edges [23–25]. Other work has shown that tetrahydrofuran (THF), an organic solvent used for SAM formation [1–2,4,6–8], can roughen the gold surface at atomic step edges [23]. We were thus interested in examining whether the loss of gold we had observed while attempting to surface-functionalize thin gold films with NHC monolayers could be attributed to the nature of the surface ligand, the solvent, or both. We therefore synthesized four different NHC-related species (Figure 1) and prepared solutions in THF, DCM, and toluene in which we immersed gold-coated glass slide tokens. We additionally exposed selected solutions to gold nanoparticles in aqueous solutions.

## Results and Discussion

**Figure 2:**
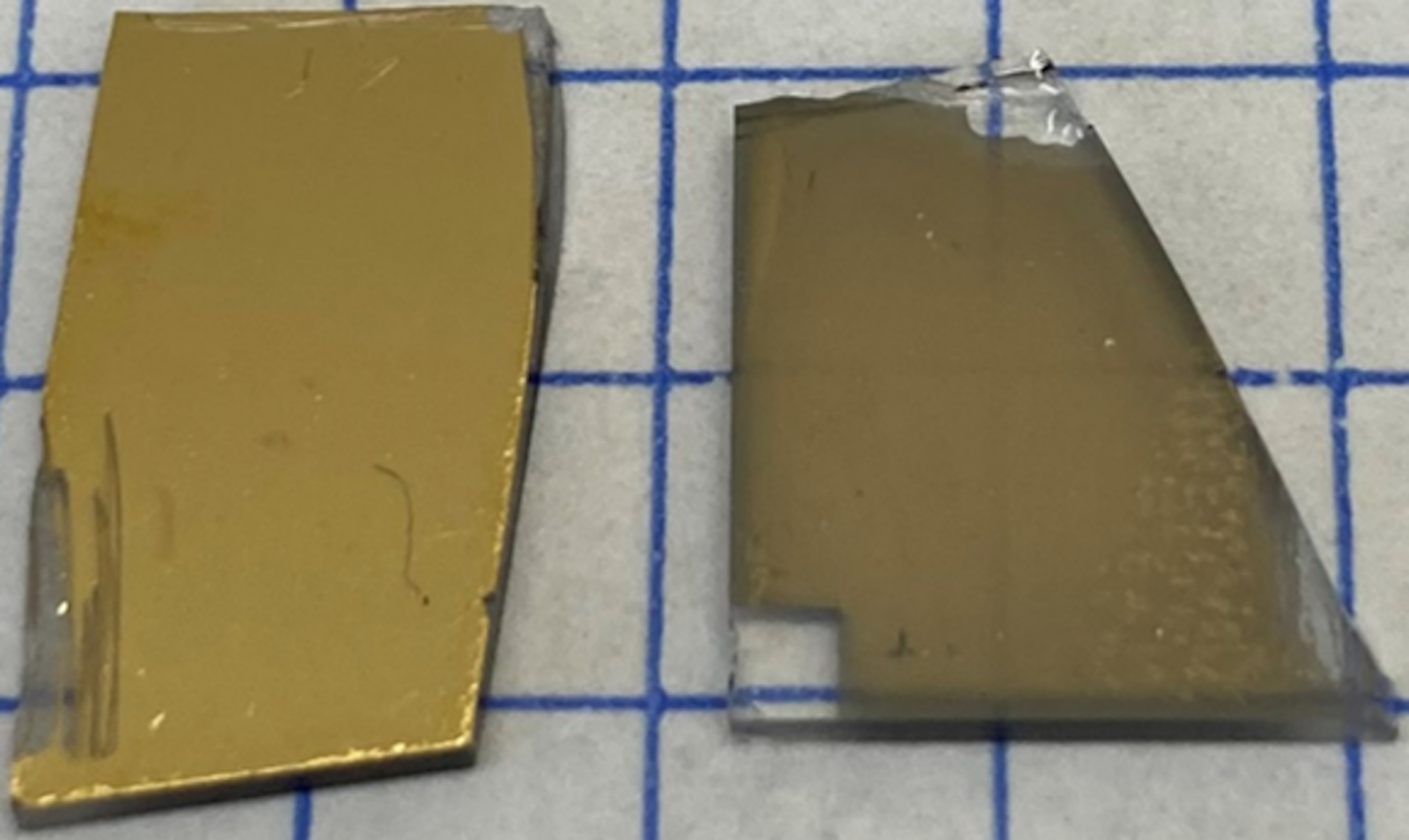
Photograph of examples of gold-coated tokens before (left) and after (right) immersion for 2 h in a 3.60 × 10^−2^ M solution of **1** in DCM. Scratches were introduced by handling, and the small gold-free rectangle was present on the as-supplied glass slides.

**Figure 3:**
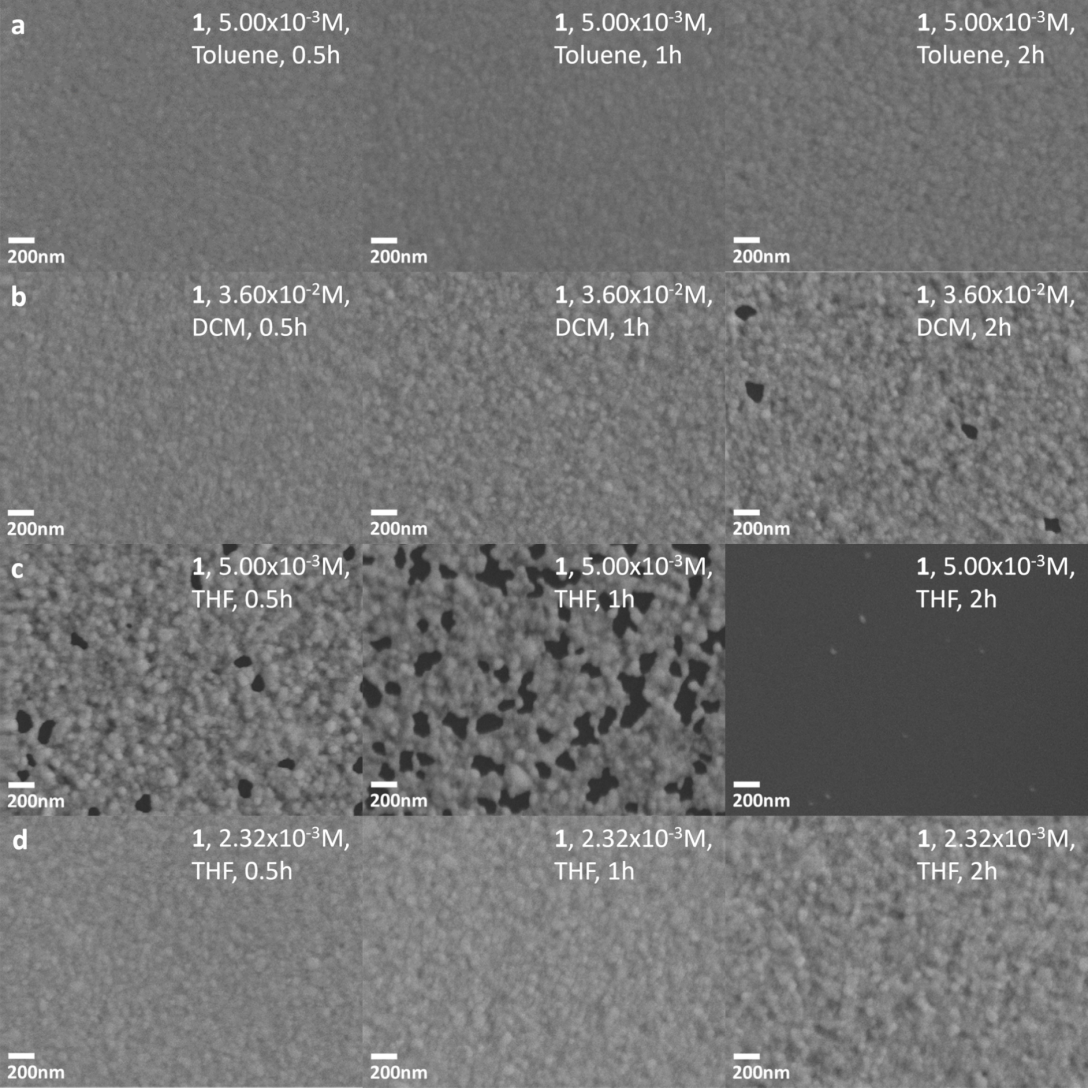
The scanning electron micrographs show the tokens after immersion in solutions of **1** in different solvents and for different durations. From left-to-right by column, the immersion times were 30 min, 1 h, and 2 h. The solvents and concentrations of **1** were (a) toluene (5.00 × 10^−3^ M), (b) DCM (3.60 × 10^−2^ M), (c) THF (5.00 × 10^−3^ M), and (d) THF (2.32 × 10^−3^ M). In (a–c), the concentrations were chosen to be close to the solubility limit of **1** in each solvent.

The photograph in Figure 2 shows the loss of gold after immersion for 2 h of a gold-coated token in a solution of **1** in DCM. There is evident loss of optical density accompanied by a transition from a reflective gold surface to one with more transmission, which allows for increased optical access to the underlying chromium adhesion layer. Figure S1 (Supporting Information File 1) shows similar results after exposure of gold-coated tokens to **1** and **2** in THF and DCM. Figure S2 (Supporting Information File 1) is a control for any potential effect of solvent. The areal contiguity of the film was unchanged after the longest solvent-only immersion time we used. This observation contrasts with the localized depletion of gold during etching by solutions of **1** in DCM and THF, as shown in the electron micrographs in Figure 3b,c. The formation of voids in the gold film allowed for comparison of this material loss between different samples without the need to standardize scanning electron microscope instrument and image settings across samples, and indeed the comparison between process conditions is centered on this prevailing result. The degree of gold loss varied with solvent, reagent concentration, and exposure time. In Figure 3 we show the effect of solutions of **1** in toluene, THF, and DCM at three different time points, near the solubility limit of **1** in each solvent, and at a lower concentration of **1** for the most aggressive formulation. In the solution of **1** in toluene (Figure 3a), for example, there is no visible change to the gold film structure compared to the controls, including as-supplied unexposed slides, in Figure S2 (Supporting Information File 1). Indeed, electron micrographs in Figure S3 (Supporting Information File 1) show no apparent loss of gold film density of any of **2**–**4** in toluene, although the low concentrations (approximately at the limit of solubility) of **2** and **3** must also be considered as a contributing factor. Similarly, Figure S4 (Supporting Information File 1) shows no apparent gold film loss in a solution of triethylamine and potassium iodide in acetone (chosen instead of THF or DCM to allow for dissolution of KI) tested as a control for the effect of Brønsted or Lewis acidity of the nitrogen atoms. In contrast, the electron micrographs in Figure 3b and Figure 3c revealed a loss of areal gold coverage by **1** in DCM and THF, respectively. Etching by **1** in DCM was evident in Figure 3b only after the longest exposure time of 2 h, whereas it was already apparent after only 30 min of exposure at a ca. seven times lower concentration in THF in Figure 3c. After 2 h of treatment with this THF-based etchant, electron microscopy revealed no trace of remaining gold film. Halving of the concentration of **1** in THF significantly reduced the degree of etching observed in Figure 3d.

**Figure 4:**
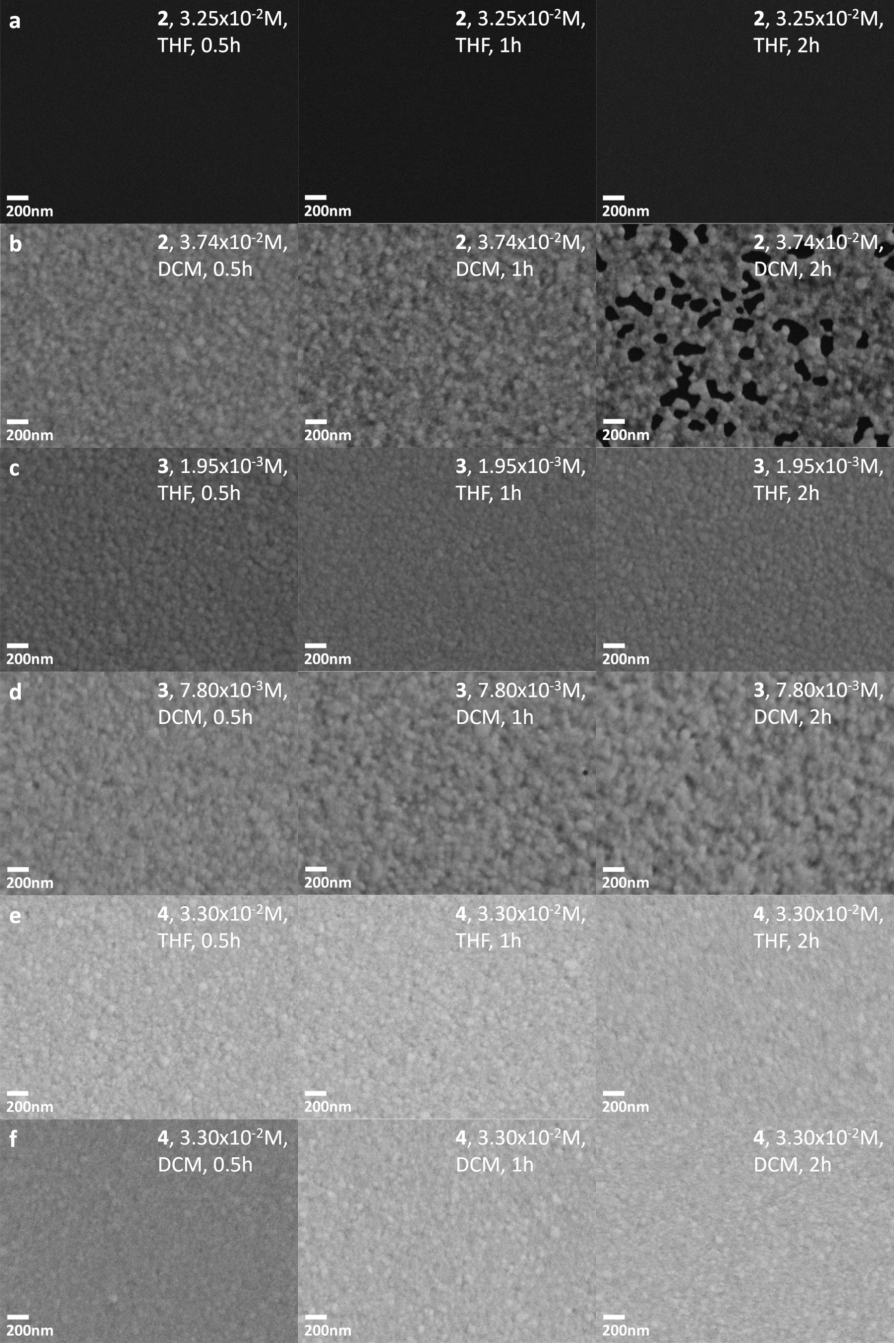
Scanning electron micrographs of tokens after immersion in solution of **2** in (a) THF (3.25 × 10^−2^ M) and (b) DCM (3.74 × 10^−2^ M), **3** in (c) THF (1.95 × 10^−3^ M) and (d) DCM (7.80 × 10^−3^ M), and **4** in (e) THF (3.30 × 10^−2^ M) and (f) DCM (3.30 × 10^−2^ M). From left to right, immersion times were 30 min, 1 h, and 2 h. An EDS spectrum (not shown) indicates that chromium, used as an adhesion layer underneath the original gold film, was present on the surface in (a).

Figure 4 shows the effect of species **2**–**4** in solutions of THF and DCM. Species **2** at a concentration of 3.25 × 10^−2^ M in THF in Figure 4a shows the most aggressive etching across species **1**–**4** in all solvents. After only 30 min there was no evidence by SEM of extant gold film. An EDS spectrum (not shown) taken after the same treatment as that in Figure 4a revealed underlying chromium. At a slightly higher concentration of **2** in DCM (3.74 × 10^−2^ M), however, the rate of etching was significantly reduced compared to the THF solution of **2**. The rate of etching of this DCM solution of **2** was closest to, but still slower than, **1** dissolved in THF to a more dilute concentration of 5.00 × 10^−3^ M. Species **3** and **4** showed insignificant or no apparent etching in both THF and DCM (as well as in toluene, as discussed regarding Figure S3 (Supporting Information File 1)). Selected EDS maps in Figure S5 (Supporting Information File 1) support that the black sections of the SEM images in Figure 3 and Figure 4 correspond to the absence of gold, that is, to complete loss of gold to the action of the etchant solution.

**Figure 5:**
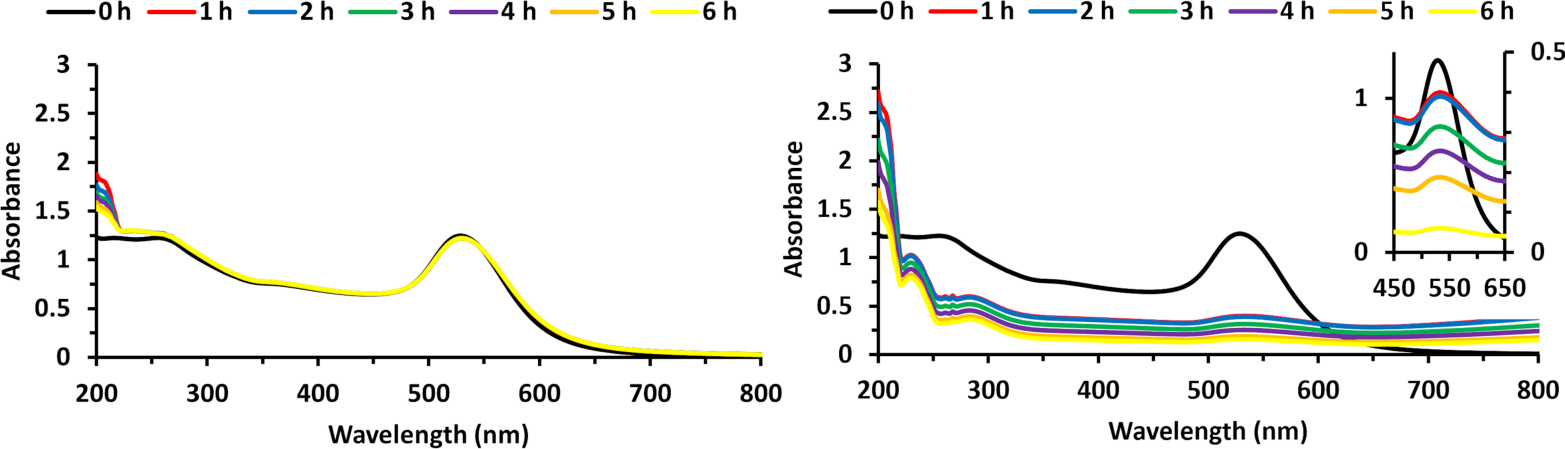
UV–vis spectra of 10 µL of 5 mM **1** in THF mixed with 10 mL of AuNPs (left) and 10 μL of 5 mM **2** in THF mixed with 10 mL of AuNPs (right) over 6 h of hourly measurements. The inset shows the peak at approx. 530 nm with the 0 h data corresponding to the left absorbance axis and the subsequent time point data corresponding to the right absorbance axis.

We mixed, with 10 min of vortexing, citrate-capped AuNPs in 10 mL aqueous solution with 10 μL of 5.00 × 10^−3^ M of **1** in THF. No appreciable change in the UV–vis spectrum was observed over 6 h of hourly measurements (Figure 5), indicating no detectable change in diameter (ca. 47 nm) and concentration of the nanoparticles [26]. This is not surprising given the low concentration of **1** and the concentration dependence shown in Figure 3c and Figure 3d. We repeated this test using a 10 μL aliquot of 5.00 × 10^−3^ M of **2** in THF. The constancy of λ_max_ = 530 nm indicated that any change in the nanoparticle size distribution was undetectable on the timescale of the measurement. Changes in nanoparticle concentration, however, were readily detected. Within the first hour of exposure, there was a ca. 75% decrease in absorbance, and there was a continued decline over the remaining 5 h. While the complete removal of the 50 nm thick gold coating of the token in Figure 4a occurred after only 30 min, that was at a substantially higher concentration of **2** in THF. In terms of generalizability, though, we are able to clearly show that etching of gold occurs from both thin films and nanoparticles, and in a single organic phase and a mixed organic and aqueous phase.

## Conclusion

The formation of molecular monolayers on gold surfaces supports a wide range of fundamental science and technological applications. While thiol-containing species continue to dominate monolayer formation, the pursuit and characterization of monolayer precursors with superior performance has been ongoing. NHCs, for example, are often proposed as a candidate with advantageous chemical stability [16]. The choice of the preparative route of the monolayers remains far less uniform than in the domain of thiol SAM formation, including solution-based approaches and melt-induced film formation [13–15,19]. In the present work we show that one must carefully consider the particular NHC-related species, the solvent, the exposure time, and the concentration in order to avoid deleterious effects such as dissolution of the gold film or nanoparticle substrate. At the same time, we introduce a molecular etchant framework for gold whose efficacy can be tuned by chemical composition, solvent composition, exposure time, and concentration.

Conventional gold etchants are predominated by inorganic chemical composition, including aqua regia (3 M:1 M hydrochloric acid to nitric acid), potassium cyanide, and triiodide [27]. More recently, however, there has been an important and active effort to develop new organic ligands that support noble metal dissolution or extraction in more environmentally benign ways [28–30]. We present here a number of single-solute gold etchants where etching performance can be tuned by judicious choice of organic species, solvent, and concentration, and where the intersection of NHCs with stable monolayer formation affords much space for further enquiry. Dissolution that occurs with lateral inhomogeneity has interesting possible application areas including the creation of porous gold without the need for Au/Ag alloys. Porous gold, itself, provides a gold film with high surface area that can be advantageous for energy conversion, catalysis, and electrochemical sensing [9,31].

## Experimental

Compounds **1** and **3** were prepared based on previously reported work [15,19], with **3** prepared by deprotonation of **1** with potassium hexamethyldisiloxane (KHMDS) in the presence of chloro(dimethyl sulfide)gold(I) under air-free conditions. Compounds **2** and **4** were also synthesized according to previous work [13–14,32]. Detailed materials and methods alongside characterizations are provided in Supporting Information File 1. Gold-coated glass slides were purchased from Electron Microscopy Sciences (71892-05, Hatfield, PA, USA). The films were 50 nm of gold deposited by electron beam evaporation onto a 2–7 nm chromium adhesion layer. Slides were cut into ca. 1 × 1 cm^2^ tokens and immersed in 2 mL of the relevant test solution in 20 mL glass vials. Vials were capped and left at room temperature in a fume hood for the allotted time of the experiment. The tokens were then removed from the test solution and rinsed in the respective organic solvent of the test solution before drying under a stream of 99.998% nitrogen gas. Electron micrographs were recorded using the SE2 detector of a Zeiss SIGMA VP field emission scanning electron microscope (SEM). Energy dispersive X-ray spectroscopy (EDS) spectra were obtained for each sample using an Oxford Instruments X-max 50 mm^2^ EDS attachment. Peaks were identified using the EDS software AZtec from Oxford Instruments (Concord, MA, USA). Gold nanoparticles were synthesized following a modified Fren’s method [33]. Raman spectra were collected using a Snowy Range Instruments Sierra 2.0 spectrometer with laser excitation at 785 nm at a laser power of 74.1 mW and an integration time of 30 s. UV–vis spectra were collected using UVProbe 2.51 software on a Shimadzu UV-3600 Plus UV–vis–NIR spectrophotometer with a 1 cm path length quartz cuvette.

## Supporting Information

File 1 Additional figures and a detailed experimental procedure and analytical characterizations.
