# Supplementary material for: N-Heterocyclic carbene-based gold etchants
Source: Beilstein J Nanotechnol. 2023 Aug 21;14:865–71. doi: 10.3762/bjnano.14.71 (PMC10477970; doi:10.3762/bjnano.14.71)
Supplement: File 1 — Additional figures and a detailed experimental procedure and analytical characterizations. [file Beilstein_J_Nanotechnol-14-865-s001.pdf]

BEILSTEIN JOURNAL OF NANOTECHNOLOGY

Supporting Information

for

N-Heterocyclic carbene-based gold etchants

Robert B. Chevalier, Justin Pantano, Matthew K. Kiesewetter and Jason R. Dwyer

Beilstein J. Nanotechnol. **2023,** *14,* 865–871. doi:10.3762/bjnano.14.71

Additional figures and a detailed experimental procedure and analytical characterizations

License and Terms: This is a supporting information file under the terms of the Creative Commons Attribution License (https://creativecommons.org/licenses/by/4.0). Please note that the reuse, redistribution and reproduction in particular requires that the author(s) and source are credited and that individual graphics may be subject to special legal provisions.
The license is subject to the *Beilstein Journal of Nanotechnology* terms and conditions: (https://www.beilstein-journals.org/bjnano/terms)

Table of Contents

Figures S2

Materials and methods S7

Overview S7

Synthesis of 1-isopropyl-5/6-nitrobenzimidazole S8

Synthesis of **1** (1,3-diisopropyl-5-nitrobenzimidazolium iodide) S8

Synthesis of **2** (1,3-diisopropyl-5-nitrobenzimidazolium hydrogen carbonate) S8

Synthesis of **3** (bis(1,3-diisopropyl-5-nitrobenzimidazolium)gold(I) iodide) S9

Synthesis of **4** (1,3-diisopropyl-5-nitrobenzimidazole-2-ylidene) S10

Synthesis of gold nanoparticles S10

NMR Spectra S11

References S12

Figures

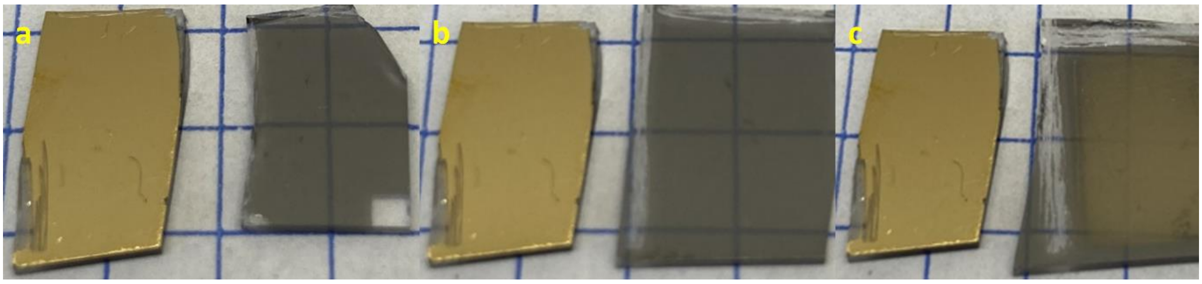

Figure S1: Gold-coated glass slide tokens soaked for 2 h in (a) 5.00×10^{-3} M of **1** in THF, (b) 3.74×10^{-2} M of **2** in DCM, and (c) 3.25×10^{-2} M of **2** in THF. In each case, an as-supplied Au-coated glass slide token is included at left for comparison and to account for any lighting variability.

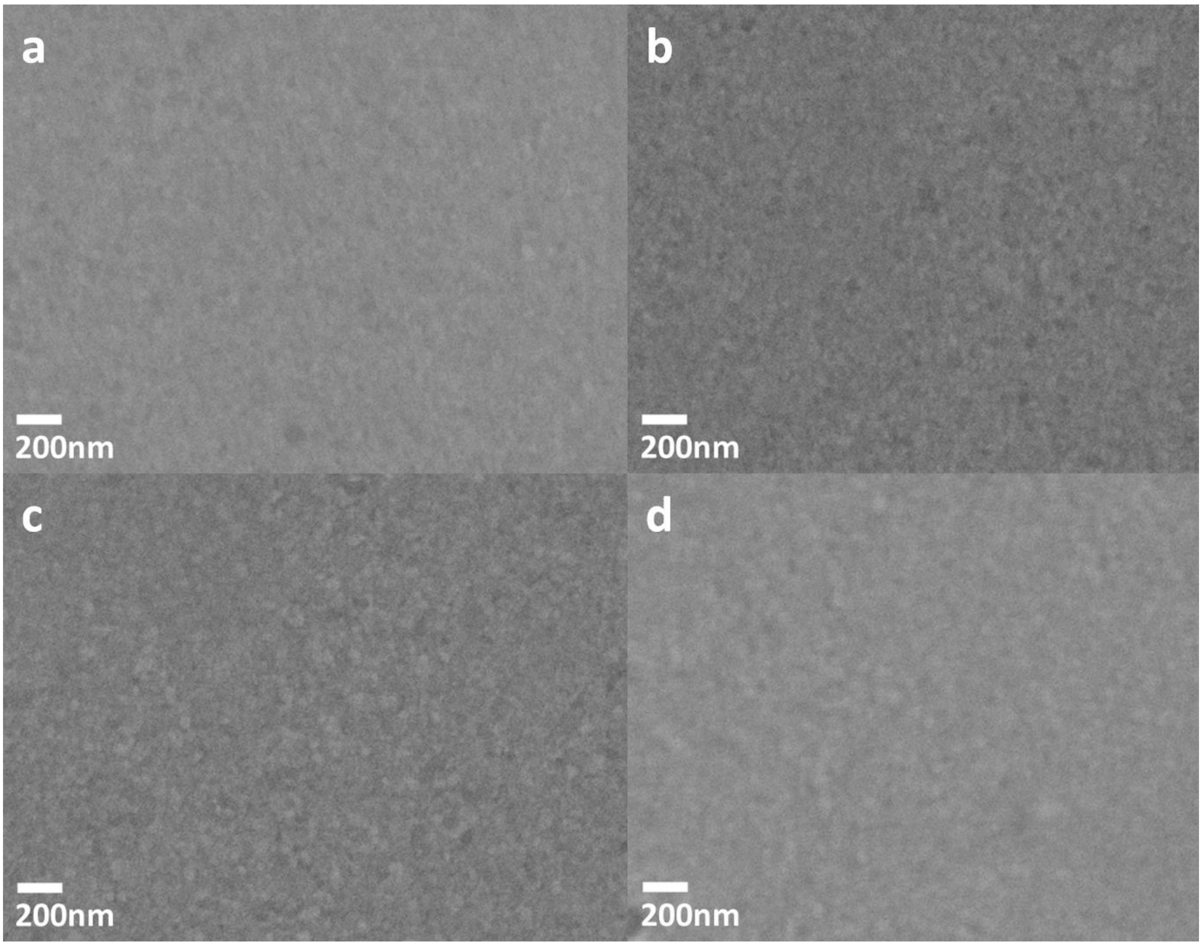

Figure S2: SEM images of Au-coated glass slide tokens (a) as-supplied and after soaking for 2 h in (b) THF, (c) DCM, and (d) toluene.

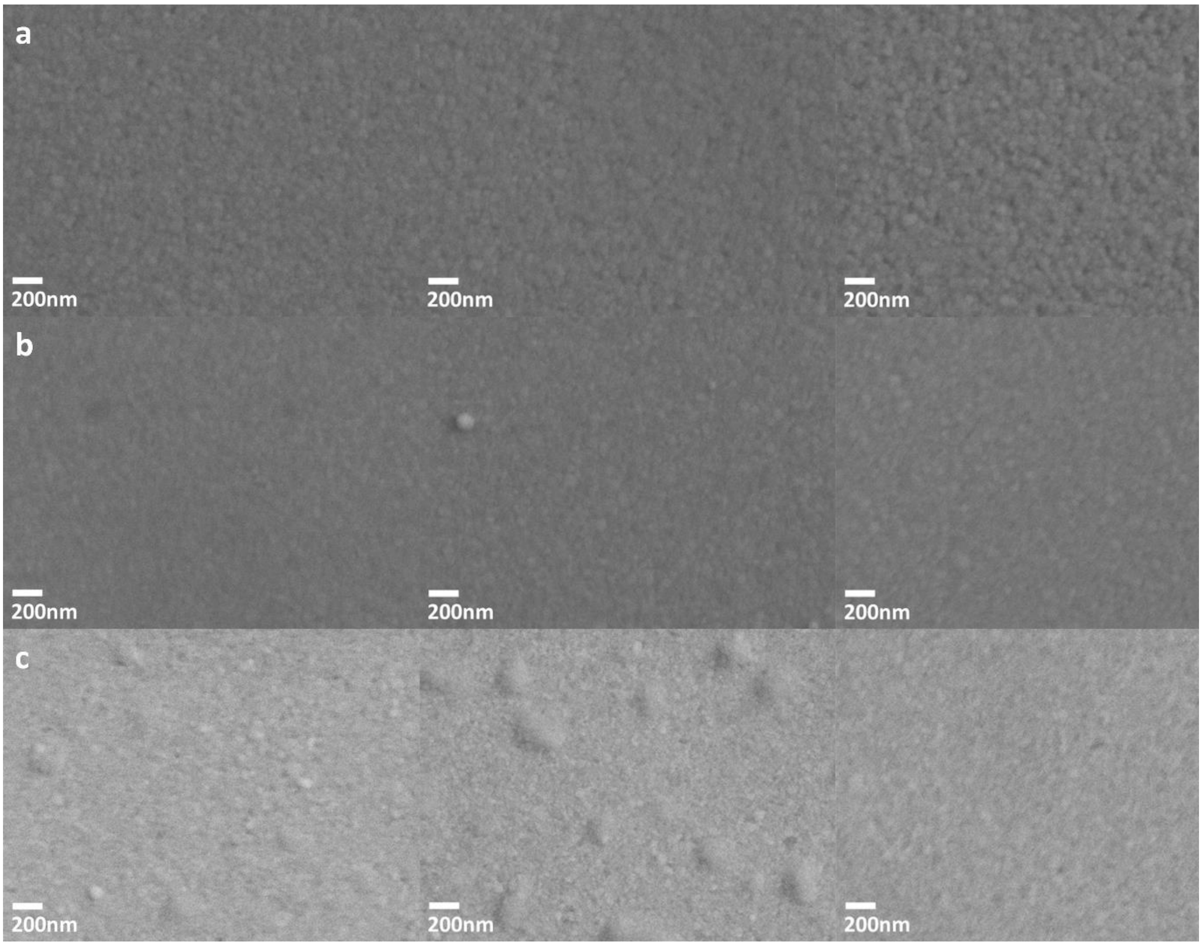

Figure S3: Electron micrographs of gold-coated tokens after immersion for (left to right) 30 min, 60 min, and 2 h in (a) 7.30×10^{-4} M of **2** in toluene, (b) 3.35×10^{-4} M of **3** in toluene, and (c) 3.30×10^{-2} M of **4** in toluene. Species concentrations were approximately at the limits of solubility in each solvent.

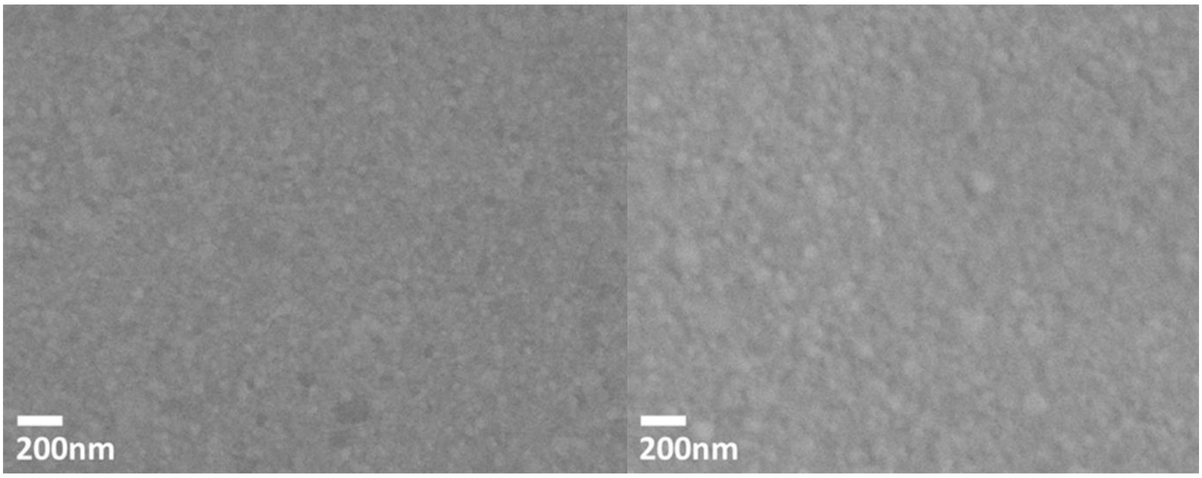

Figure S4: SEM images of Au-coated glass slide tokens soaked for 2 h in acetone (left) and in an equimolar 3.74 × 10^{-2} M mixture of triethylamine (TEA) and potassium iodide (KI) in acetone (right).

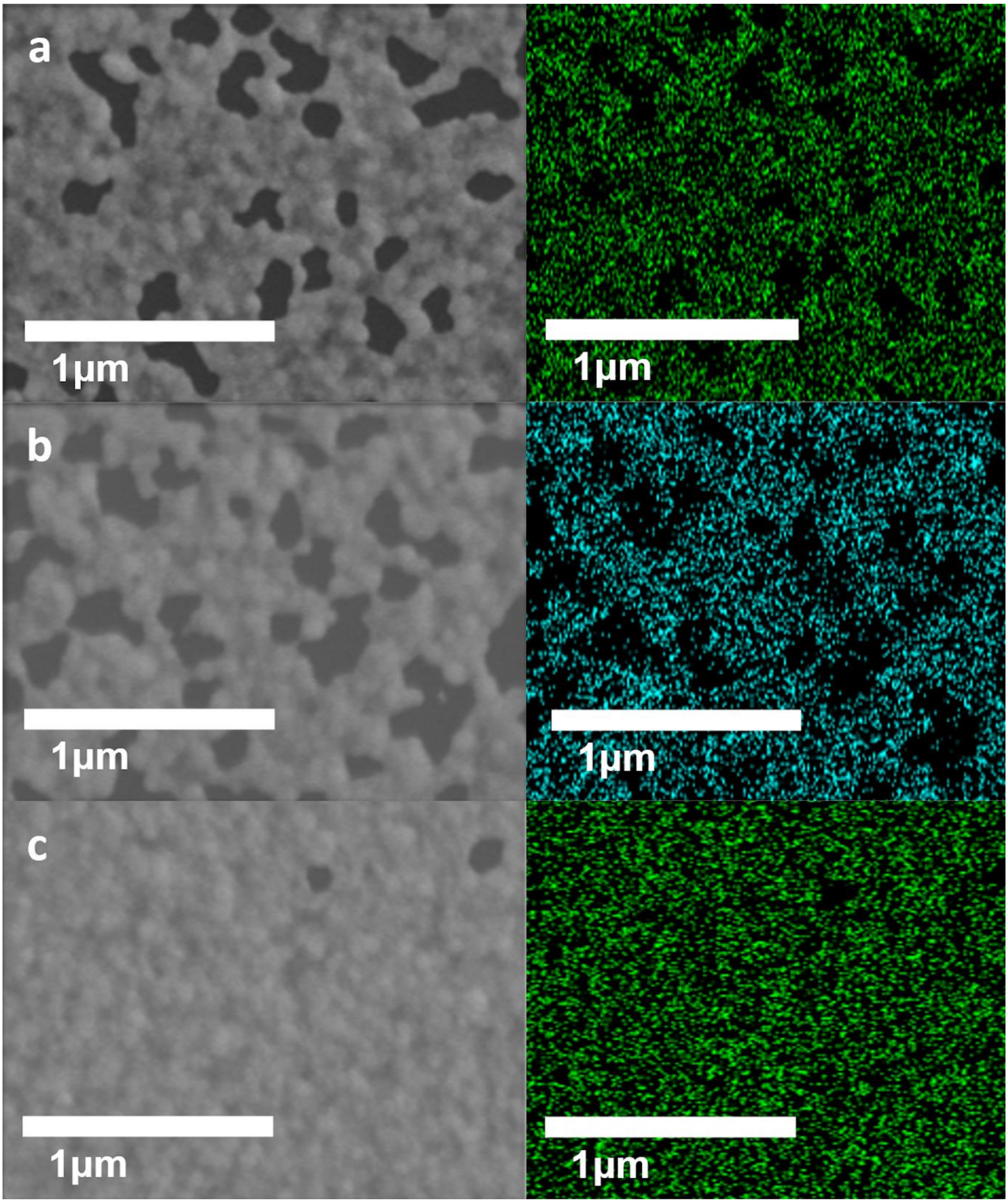

Figure S5: Selected SEM regions of interest (left) and corresponding EDS maps for gold (right) for (a) **1** in DCM (3.60×10^{-2} M, 2 h); (b) **1** in THF (5.00×10^{-3} M, 1 h); and (c) **2** in DCM (3.74×10^{-2} M, 2 h).

Materials and methods

Overview

All air-free chemistry was performed using standard Schlenk techniques under N_2, and all workups were conducted under ambient conditions, unless noted otherwise. All chemicals were purchased from Fisher Scientific (Pittsburgh, PA, USA) and used as received, unless noted otherwise. Acetone (329000000, 100%) was purchased from Pharmaco (Charlotte, NC, USA). Dichloromethane (D37-4, ≥99.5%), tetrahydrofuran (360589 ≥99.0%, BHT inhibitor), and toluene (T324SK-4, 99.9%) for air-free reactions were dried via an Innovative Technology solvent purification system. Anhydrous methanol (322415, 99.8%), potassium hexamethyldisiloxane (KHMDS) (324671, 95%) and chloro(dimethyl sulfide)gold(I) (420727, no purity given) were purchased from Sigma-Aldrich (Burlington, MA, USA). 5-Nitrobenzimidazole (N0152, >98.0%) and 2-iodopropane (I0069, >99.0%) were purchased from TCI (Portland, OR, USA). Cesium carbonate (12887, 99.0%) was purchased from Alfa Aesar (Haverhill, MA, USA). Methanol-d_4 (DLM-24-50, 99.8%), dimethyl sulfoxide-d_6 (DLM-10-100, 99.9%), and chloroform-*d* (DLM-7-100, 99.8%) were purchased from Cambridge Isotope Labs (Tewksbury, MA, USA) and dried with 4 Å molecular sieves prior to use for NMR characterization. NMR spectra were taken on a Bruker Ascend 400 MHz spectrometer at 298 K. All ^{1}H and ^{13}C NMR spectra were referenced to solvent residual signals. Type-I water (ca. 18 MΩ·cm) was obtained from a Millipore Synergy UV (Billerica, MA) and was used for experiments. Glass vials were purchased from Fisher Scientific (Pittsburgh, PA, USA), and 99.995% N_2 was purchased from Airgas (Radnor, PA, USA).

Synthesis of 1-isopropyl-5/6-nitrobenzimidazole

To a 3-necked round bottom flask fitted with a reflux condenser, septum with N_2 inlet and a septum and charged with a magnetic stir bar, 5-nitrobenzimidazole (3.0301 g, 18.57 mmol), cesium carbonate (6.0028 g, 18.50 mmol), 2-iodopropane (3.30 mL, 33.06 mmol), and acetonitrile (100 mL) were added. The vessel was purged with N_2 and refluxed overnight under N_2. The reaction mixture was cooled then diluted with 100 mL ethyl acetate and washed with water (3 × 30 mL), followed by one wash with brine (30 mL). The combined organic layers were dried with sodium sulfate, filtered, then concentrated under reduced pressure, resulting in a pale yellow solid as a mixture of isomers (3.4580 g, 91 %). Characterization matched the literature [1].

Synthesis of 1 (1,3-diisopropyl-5-nitrobenzimidazolium iodide)

To a round bottom flask equipped with a reflux condenser and magnetic stir bar, 1-isopropyl-5/6-nitrobenzimidazole (1.867 g, 9.10 mmol) and 2-iodopropane (18.5 mL, 185.3 mmol) were added, then heated at 90 °C for 60 h under N_2. After 60 h, the mixture was diluted with 15 mL toluene, then filtered. The solid product was then dissolved in methanol (ca. 6 mL), and to this was added diethyl ether until a yellow precipitate formed (2.0784 g, 61%). Characterization matched the literature [1].

Synthesis of 2 (1,3-diisopropyl-5-nitrobenzimidazolium hydrogen carbonate)

To an oven-dried Schlenk flask equipped with magnetic stir bar, 1,3-diisopropyl-5-nitrobenzimidazolium iodide (379.0 mg, 1.01 mmol) and sodium bicarbonate (88.6 mg, 1.05 mmol) were added, then the flask was purged with N_2 and sealed with a septum. Anhydrous methanol (4 mL) was added via syringe, and the mixture was stirred under N_2 for 48 h. After 48 h, the reaction mixture was filtered through Celite. The Celite was

then washed with excess methanol to ensure complete transfer. The filtrate was added to diethyl ether to precipitate a yellow solid (275.1 mg, 88%) [2].

To test for the disappearance of iodide, a silver nitrate test was performed. A small amount of the product was dissolved in ca. 1 mL water, and to one drop of this was added excess aqueous silver nitrate (1 M). A white precipitate of silver bicarbonate formed, which turned colorless upon addition of nitric acid. This is indicative of complete exchange of iodide [3].

^{1}H NMR (400 MHz, methanol-d_4) δ 8.90 (d, J = 2.0 Hz, 1H), 8.45 (dd, J = 9.2, 2.1 Hz, 1H), 8.24 (d, J = 9.2 Hz, 1H), 5.14 (dhept, J = 20.3, 6.7 Hz, 2H), 1.71 (dd, J = 6.7, 2.3 Hz, 12H).

^{13}C NMR (101 MHz, MeOD) δ 160.02, 146.33, 142.03, 134.75, 131.19, 121.64, 115.09, 110.60, 52.57, 52.38, 21.10, 20.94.

Synthesis of 3 (bis(1,3-diisopropyl-5-nitrobenzimidazolium)gold(I) iodide)

In a glove box, KHMDS (142.6 mg, 0.715 mmol) and 1,3-diisopropyl-5-nitrobenzimidazolium iodide (258.5 mg, 0.689 mmol) were added to an oven-dried Schlenk flask equipped with magnetic stir bar. Under N_2 flow attached to a Schlenk manifold, THF (20 mL) was added via syringe, and the mixture was stirred for 15 min, after which chloro(dimethyl sulfide)gold(I) (100.8 mg, 0.342 mmol) was added. This solution was stirred overnight under N_2 after which it was concentrated under reduced pressure. The residue was redissolved in DCM and hexanes was added, producing a precipitate, an off-white solid (80.8 mg, 29%). Characterization matched the literature [1].

Synthesis of 4 (1,3-diisopropyl-5-nitrobenzimidazole-2-ylidene)

In a glove box, KHMDS (57.5 mg, 0.288 mmol) and 1,3-diisopropyl-5-nitrobenzimidazolium iodide (98.3 mg, 0.262 mmol) were added to an oven-dried Schlenk flask equipped with magnetic stir bar. Under N_2 flow attached to a Schlenk manifold, DCM (8 mL) was added via syringe and stirred under N_2 overnight. Under inert conditions, the mixture was filtered via a medium-frit Schlenk tube to another oven-dried Schlenk flask, and the resulting orange solution was used without purification for further experiments [1]. Solutions of **4** in toluene and THF were prepared similarly.

Synthesis of gold nanoparticles

A modified Fren's method was used to synthesize gold nanoparticles [4]. Stock solutions (50 mL) of 38.8 mM sodium citrate dihydrate (0.5706 g) and 1.0 mM gold(III) chloride trihydrate (0.0197 g) in type-1 water were made using a 50 mL volumetric flask. To a 50 mL Erlenmeyer flask equipped with a magnetic stir bar, 20 mL of 1.0 mM gold(III) chloride trihydrate was added and stirred and heated until boiling. Once boiling, 2 mL of 38.8 mM sodium citrate dihydrate was added and boiled with stirring for 10 min. The volume was kept approximately constant by adding type-I water throughout the boiling process. After 10 min, the solution was allowed to cool to room temperature in a fume hood. UV–vis measurements were performed to determine particle size [5] in a 1 cm path length quartz cuvette from Spectrocell (Oreland, PA, USA) on a Shimadzu UV-3600 Plus UV–vis–NIR spectrophotometer using the UVProbe 2.51 software.

NMR spectra

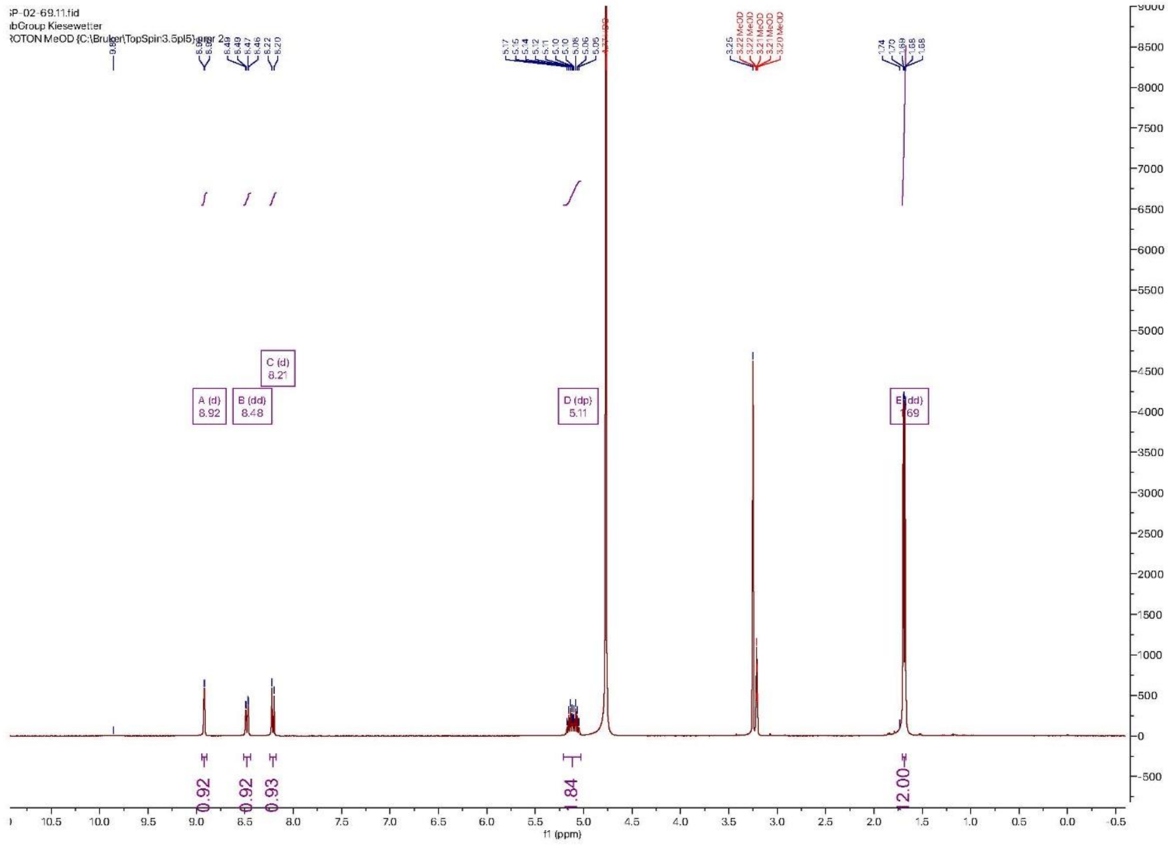

Figure S6: ^{1}H NMR spectrum (400 MHz, methanol-d_4) of **2**.

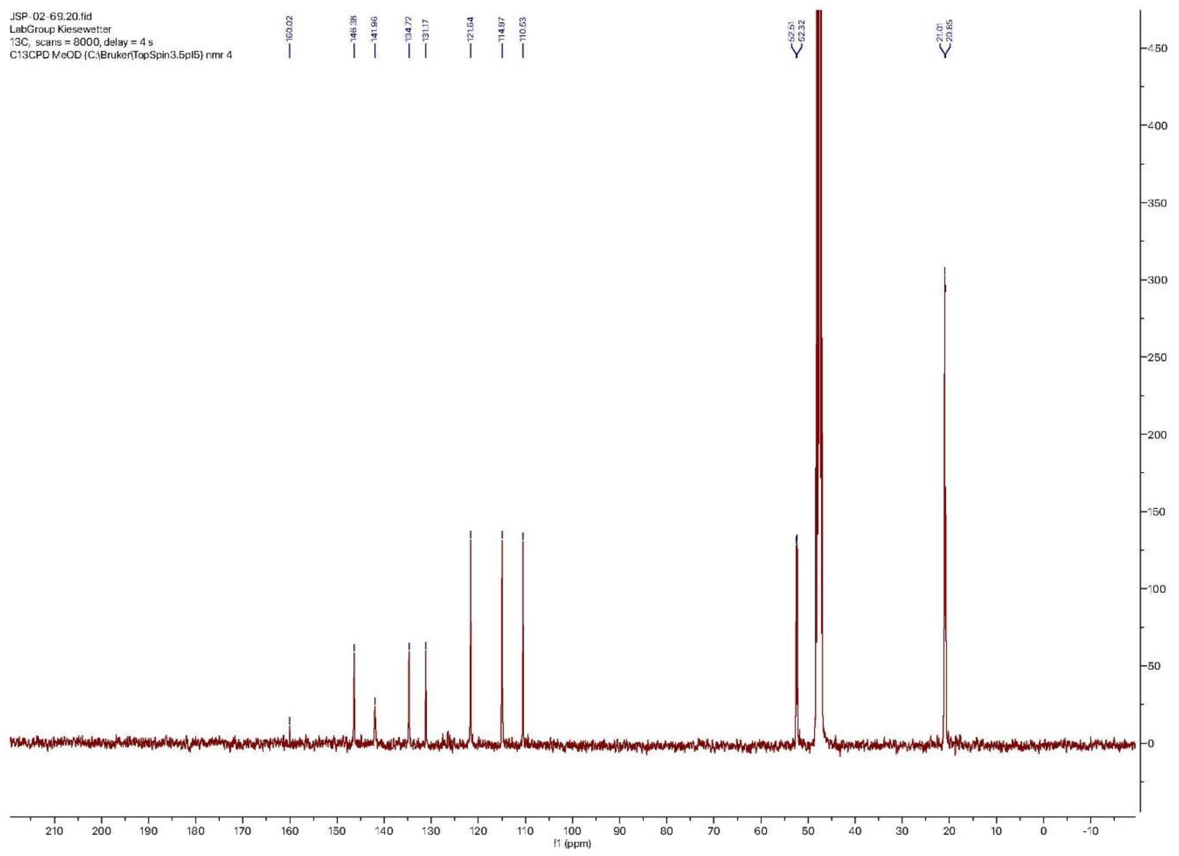

Figure S7: ^{13}C NMR spectrum (101 MHz, methanol-*d*$_4$) of **2**.

References

1. DeJesus, J. F.; Sherman, L. M.; Yohannan, D. J.; Becca, J. C.; Strausser, S. L.; Karger, L. F. P.; Jensen, L.; Jenkins, D. M.; Camden, J. P. *Angew. Chem., Int. Ed.* **2020,** *59,* 7585–7590. doi:10.1002/anie.202001440
2. Fèvre, M.; Vignolle, J.; Taton, D. *Polym. Chem.* **2013,** *4,* 1995–2003. doi:10.1039/c2py20915b
3. Crudden, C. M.; Horton, J. H.; Narouz, M. R.; Li, Z.; Smith, C. A.; Munro, K.; Baddeley, C. J.; Larrea, C. R.; Drevniok, B.; Thanabalasingam, B.; McLean, A. B.; Zenkina, O. V.; Ebralidze, I. I.; She, Z.; Kraatz, H.-B.; Mosey, N. J.;

Saunders, L. N.; Yagi, A. *Nat. Commun.* **2016,** *7,* 12654. doi:10.1038/ncomms12654

4. Frens, G. *Nature (London), Phys. Sci.* **1973,** *241,* 20–22. doi:10.1038/physci241020a0
5. Haiss, W.; Thanh, N. T. K.; Aveyard, J.; Fernig, D. G. *Anal. Chem. (Washington, DC, U. S.)* **2007,** *79,* 4215–4221. doi:10.1021/ac0702084
